# Medical Diagnosis

**Published:** 1889-04

**Authors:** 


MEDICAL DIAGNOSIS, a Manual of Clinical Methods by J. G. Brown, M. D. of Edinburgh. Second edition illustrated, p. p. 260 New York; E. B. Treat, 771 Broadway, 1888.

Any work that treats of physical diagnosis in a fair and honest manner, is a valuable book.

Dr. Brown has not gone into the subject so extensively as some others who have treated this subject, but his arrangement is good, and what is to be especially commended, he has emphasized the significance of the symptoms which are of the greatest practical value in diagnosis. B.

